# Biological characteristics and pathogenicity comparison of *Nocardia seriolae* isolated from *Micropterus salmoides* and *Channa argus*

**DOI:** 10.3389/fvets.2024.1367066

**Published:** 2024-04-10

**Authors:** Wei Zhang, Kexin Zhou, Lei Huang, Na Yang, Lingyun Lin, Lu Chen, Jiayun Yao, Mingxin Dong, Jinyu Shen, Xiaoyi Pan

**Affiliations:** ^1^School of Pharmacy, Qingdao University, Qingdao, China; ^2^Agriculture Ministry Key Laboratory of Healthy Freshwater Aquaculture, Key Laboratory of Fish Health and Nutrition of Zhejiang Province, Zhejiang Institute of Freshwater Fisheries, Huzhou, China; ^3^Ningbo Sansheng Biological Technology Co., Ltd., Ningbo, China

**Keywords:** *Nocardia seriolae*, biological characteristics, pathogenicity, homogeneity, *Micropterus salmoides*, *Channa argus*

## Abstract

*Nocardia seriolae* is the primary pathogen causing nocardiosis in various fish species, leads to significant economic losses in the aquaculture industry. In this study, 10 bacterial strains isolated from *Micropterus salmoides* and *Channa argus* infected with nocardiosis, were identified as *N. seriolae* by physiological and biochemical identification, as well as 16S rDNA sequencing. Moreover, the key virulence-related genes such as ESX-1, T7SS-2, T7SS-3, EspG1, sodC, sod2 and ESAT6 were all positive, and showing high homology among different strains. Pathogenicity testing revealed mortality rates ranging from 70 to 100%, accompanied by the presence of white nodules in the viscera of deceased fish. The drug sensitivity test demonstrated that LY21811, the most lethal strain, exhibited high sensitivity to nine types of antibiotics, including azithromycin, doxycycline, florfenicol and compound sulfamethoxazole, yet showed complete resistance to β-lactam antibiotics. Additionally, the tannic acid also demonstrated potent inhibitory effects against LY21811, with a minimum inhibitory concentration of 0.0625 mg/mL. These results showed that *N. seriolae* originated from *M. salmoides* and *C. argus* in Zhejiang Province were highly conserved, demonstrating a high homogeneity in genetic characteristics, pathogenicity and antimicrobial susceptibilities. These results provide a foundation for further research on the pathogenic characteristics and disease prevention of *N. seriolae* infections.

## 1 Introduction

Fish nocardiosis is a systemic disease that has led to substantial economic losses in both marine and freshwater aquaculture industries (1–3). Pathogenic bacteria including *Nocardia asteroids* (in neon tetras, *Hyphessobrycon innesi*) (4), *Nocardia seriolae* (in yellowtails, *Seriola quinqueruiata* and *Seriola purpurascens*) (5) and *Nocardia salmonicida* (in blueblack salmon, *Oncorhynchus nerca*) (6) have been confirmed as causative agents of fish nocardiosis. Particularly, *N. seriolae* has emerged as a predominant pathogen affecting various fish species, such as largemouth bass (*Micropterus salmoides*) and snakehead (*Channa argus*) (7–9). *N. seriolae* can infiltrate fish through wounds, gills, and feeds (10), manifesting symptoms in diseased fish that include pinpoint hemorrhages, ulcerative lesions, reddening of the snout, and nodules in the liver, spleen, kidney, peritoneum, and muscles (11). These infections caused by *N. seriolae* have been documented across multiple countries (12), exhibiting high mortality rates, with natural morbidity rates ranging from 20 to 60% and mortality rates as high as 100% (13).

*Nocardia,* as reported in many studies, has been found to be catalase-positive, oxidase-negative, and capable of reducing nitrate and aesculin, but not degrading xanthine, tyrosine, casein, starch, and gelatin. It can utilize citrate as the sole carbon source but not mannitol, arabinose, and sorbitol. These biochemical characteristics can be used as methods for identifying *Nocardia* species (6, 14, 15).

Understanding the mechanisms behind *Nocardia* virulence is crucial for developing effective therapeutic strategies against nocardiosis. The virulence of *Nocardia* is primarily determined by a set of key virulence genes that encode proteins involved in various pathogenic processes (16–18). Among these genes, ESX-1, T7SS-2, T7SS-3, EspG1, sodC, sod2, and ESAT6 have been extensively studied for their roles in *Nocardi*a pathogenicity, enabling this bacterium to survive and thrive within host organisms (19–23). Additionally, researchers utilized whole-genome sequencing, referencing virulence factors from *Mycobacterium* and human-derived *Nocardia*, to predict and disclose virulence factors of *N. seriolae*. They identified genes encoding proteins akin to hemolysins and toxin proteins from Vibrio in *N. seriolae* isolated from *Anguilla japonica* (24, 25). These discoveries establish a basis for further investigations into the virulence factors of *N. seriolae*.

Largemouth bass has been an important commercial cultured fish in China, and Zhejiang province is one of the most important culturing area. However, intensive or semi-intensive practices in largemouth bass farming have substantially increased the risk of contagious diseases. Outbreaks of contagious diseases, characterized by skin ulceration and nodular lesions in internal organs, have been reported from almost every year from April to October in various regions.

To investigate the differences among strains of *N. seriolae* isolated from largemouth bass and snakehead, this experiment analyzed the biological characteristics, virulence genes, pathogenicity, and drug sensitivity of 10 dominant strains obtained from fish afflicted with “nodular disease” in Zhejiang Province. This study aims to explore the biological distinctions among these various strains and provide insights for understanding and guiding the prevention and control of fish nocardiosis.

## 2 Materials and methods

### 2.1 Experimental fish and strains

Healthy largemouth bass (*M. salmoides*) used in the experiment, weighing a standard 20.0 g ± 2.0 g, were supplied by the Comprehensive Experimental Base of Zhejiang Institute Freshwater Fisheries. Prior to the experiment, these fish underwent temporary rearing for 1 week in a circulating aquaculture system at 25°C and received daily feeding with commercial pellets designed for largemouth bass. Additionally, five randomly selected largemouth bass underwent screening for *N. seriolae*, largemouth bass ranavirus (LMBV), and infectious spleen and kidney necrosis virus (ISKNV) to confirm the absence of pathogens among the experimental fish (8, 26).

Ten strains of *N. seriolae*, obtained from diseased largemouth bass and snakehead in China, were employed in this experiment. Detailed information regarding these strains is provided in Table 1.

**Table 1:** Information of *N. seriolae* strains.

Strain No.	Strain	Date of sampling	Infected object	Location
1	WL171012	2017-10-12	*C. argus*	Deqing, China
2	LY20810	2020-08-10	*M. salmoides*	Linghu, China
3	LY20910	2020-09-04	*M. salmoides*	Linghu, China
4	LY21712	2021-07-08	*M. salmoides*	Wuxi, China
5	LY21811	2021-08-07	*M. salmoides*	Nanxun, China
6	LY21930	2021-09-30	*M. salmoides*	Nanxun, China
7	LY211019	2021-10-19	*M. salmoides*	Deqing, China
8	LY211026	2021-10-26	*M. salmoides*	Deqing, China
9	LY211129	2021-11-29	*M. salmoides*	Deqing, China
10	WL22722	2022-07-22	*C. argus*	Wuxi, China

### 2.2 Physiological and biochemical identification

The physiological and biochemical properties of the 10 strains were determined by employing bacterial biochemical identification tubes (manufactured by Hangzhou Microbiological Reagent Co., Ltd.), following the identification criteria outlined in the “Bergey’s Manual of Systematic Bacteriology” and the methodologies described for *N. seriolae* in the related literature (9, 27). Various enzymatic activities such as catalase, oxidase, urease, and nitrate reduction, along with hydrolytic activities including esculin, gelatin, starch, and casein, were evaluated for these strains. Additionally, their capability to utilize specific carbon sources such as mannitol, arabinose, sorbitol, and citrate was also investigated.

### 2.3 Phylogenetic analysis

DNA from the tested bacterial strains was extracted using the TIANamp Bacteria DNA Kit (Tiangen Biotech Co., Ltd.). Subsequently, the bacterial DNA served as a template for PCR amplification utilizing universal 16S rDNA primers (Table 2). The PCR products were sequenced by Hangzhou Shangya Biotechnology Co., Ltd. Phylogenetic trees were constructed using the Neighbor-Joining method with 1,000 bootstrap replicates in MEGA-X software. Intra-species similarities were analyzed using Lasergene/MegAlign software.

**Table 2:** Primer sequences and PCR amplification conditions in this study.

Primer name	Sequence (5′–3′)	Sequence length (bp)	Extending time (s)	Annealing temperature (°C)	GeneID in accession nz_ap017900
*16S rDNA*-F	AGAGTTTGATCCTGGCTCAG	1,500	100	55	/
*16S rDNA*-R	GGTTACCTTGTTACGACTT				
*MY16S*-F	AGAGTTTGATCCTGGCTCAG	1,030	30	58	61145434
*MY16S*-R	TGCACACAGGCCACAAGGGA				
*ESX-1*F	ATTCGGCACTCGCATGTCC	482	15	59	61149338
*ESX-1*R	CAGCGGCAGATCGGGAAT				
*T7SS-2*F	GCCCATATCAACCGAATCC	436	15	58	61152378
*T7SS-2*R	CGGTGCGAATCATGTTGTG				
*T7SS-3*F	TCGTGAACTCTTCGCTATGCC	415	15	58	61145993
*T7SS-3*R	CCTGGTCGAACGGACTGCT				
*EspG-1*F	ACGCTGTCACTGGACGAAATG	750	15	58	61145075
*EspG-1*R	TCGCAGCGGCAGGGAT				
*SodC*F	CGGTCCGCTCTGGCATCA	787	15	58	61145409
*SodC*R	CCGGGGCGAAGAGTTGAA				
*sod2*-F	GAATGATGGTTGGGTGCG	861	30	55	61145060
*sod2*-R	GGTGCCCGTGTTGCTGTT				
*ESAT6*F	GACTCGGTCCTGAAGTGGTG	1897	30	58	61151040
*ESAT6*R	TGTCCCTGATGACTGTCTCCT				

### 2.4 Detection of virulence genes

Based on the complete genome sequence of *N. seriolae* (GenBank ID: AP017900), eight pairs of primers related to virulence genes ESX-1, T7SS-2, T7SS-3, EspG1, sodC, sod2, ESAT6, and the positive control MY16S were designed. PCR amplification was executed using the bacterial DNA as a template. Comprehensive details regarding the primer sequences and reaction conditions are outlined in Table 2.

### 2.5 Pathogenicity testing

Ten bacterial strains were individually resuscitated on brain heart infusion (BHI) agar plates. Subsequently, single colonies were carefully picked and inoculated into BHI liquid medium. They were cultured at 28°C and 150 r/min on a shaker for 6 days. Following centrifugation at 2,000 rpm for 3 min, the supernatant was discarded, and the bacterial cells were delicately homogenized in PBS. The concentration bacterial suspension was adjusted to 1.0 × 10^7^ CFU/mL and reserved for further utilization.

For the experimental phase, 220 largemouth bass (as detailed in Section 2.1) were randomly divided into 10 experimental groups and 1 control group, with 20 fish in each group. The experimental groups were each injected intraperitoneally with the bacterial suspension of the 10 strains of *N. seriolae* at a concentration of 1.0 × 10^7^ CFU/mL, with a volume of 0.1 mL per fish, while the control group injected an equivalent volume of sterile PBS. Throughout the experimental timeline, daily oxygen supplementation was ensured, and the water temperature was meticulously maintained at 27 ± 1°C. Continuous monitoring of the fish was carried out for 30 days. The mortality rate was calculated by recording the number of deaths and observing disease conditions among the largemouth bass in each group.

### 2.6 Drug sensitivity testing

In the pathogenicity test, the bacterial strain that exhibited with the highest mortality rate in largemouth bass sensitivity was subjected to sensitivity testing against 16 antibiotics and 10 phytochemicals. An aliquot of 0.1 mL of the bacterial suspension, with a concentration of 1.0 × 10^8^ CFU/mL, was uniformly spread onto solid BHI medium. Sixteen antibiotic sensitivity discs (see Table 3, Hangzhou Microbiological Reagent Co., Ltd.) were uniformly was placed equidistantly on the agar plates. After incubation at 28°C for 5 days, the diameter of the inhibition zone around each disc was precisely measured.

**Table 3:** Antimicrobial susceptibility of strain LY21811.

Drug name	Drug content (μg/tablet)	Standard diameter of inhibited zone (mm)			Sensibility	Inhibitory zone (mm)
		R	I	S		
Azithromycin	15	≤13	14–17	≥18	S	38.67 ± 0.94
Doxycycline	30	≤12	13–15	≥16	S	36.00 ± 2.30
Florfenicol	30	≤12	13–17	≥18	S	31.67 ± 2.36
Compound neomycin sulfate	23.75	≤10	11–15	≥16	S	30.67 ± 0.47
Oxytetracycline	10	≤12	13–14	≥15	S	30.00 ± 0.82
Levofloxacin	5	≤16	17–19	≥20	S	28.30 ± 0.47
Neomycin sulfate	30	≤12	13–16	≥17	S	27.67 ± 3.30
Kanamycin	30	≤13	14–17	≥18	S	24.00 ± 0.82
Clindamycin	2	≤14	15–20	≥21	S	22.00 ± 2.16
Enrofloxacin	10				I	18.67 ± 0.94
Neomycin	30	<16		≥16	I	16.00 ± 2.94
Cefalexin	30	≤14	15–17	≥18	R	—
Amoxicillin	20	<14	14–17	>17	R	—
Cefoperazone	30	≤14	15–17	≥18	R	—
Sulfamethoxazole	75	<16	16–20	>20	R	—
Penicillin	10 U	≤14		≥15	R	—

The letters R, I and S indicate resistance, middle sensitive, and sensitivity, respectively. The inhibition zone is expressed by “mean ± standard.”

For the phytochemicals, stock solutions were prepared by dissolving 100 mg of each compound-oleanolic acid, tannic acid, chlorogenic acid, coumarin, magnolol, matrine, quercetin, glycyrrhizic acid, baicalin, and andrographolide-in 1 mL of DMSO solution. Following vigorous shaking to ensure complete dissolution, the solutions were sterilized by filtration through a 0.2 μm cellulose acetate membrane. On the BHI medium, 100 μL of LY21811 bacterial suspension at a concentration of 1.0 × 10^8^ CFU/mL was uniformly spread. Wells were created in the agar using a sterile punch, and 5 μL of the prepared phytochemical solution (equivalent to 0.5 mg) was added to the respective wells. After the solutions were fully absorbed, the plates were inverted and incubated at 28°C for 5 days. Subsequently, the diameter of the largest inhibition zones produced by the phytochemicals were then measured.

The phytochemicals that produced the largest inhibition zone was further assessed using a two-fold serial dilution method to determine its minimum inhibitory concentration (MIC). The drug was diluted to concentrations of 0.25 mg/mL, 0.125 mg/mL, 0.0625 mg/mL, and 0.0313 mg/mL. The inhibition zones were measured using the same method as described above.

## 3 Results

### 3.1 Morphology and physiological-biochemical characteristics

**Figure 1:**
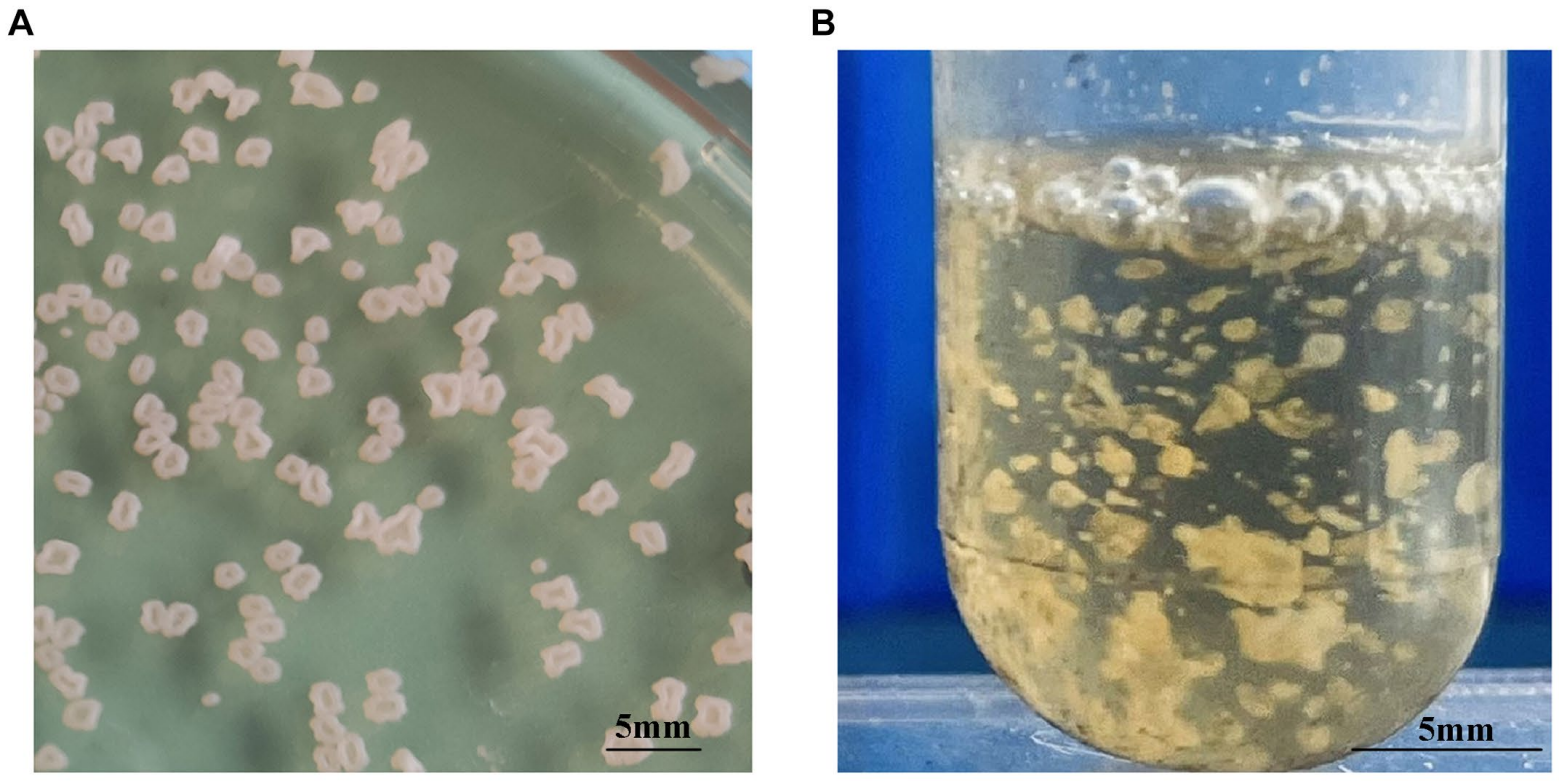
Colony morphology of *N. seriolae* strains. **(A)** Cultivated on BHI solid medium **(B)** cultivated in BHI liquid medium. In solid culture medium, the bacterial strain exhibits a dry cauliflower-like appearance. In liquid medium, the bacterial strain formed flocculent suspensions.

Each of the 10 strains outlined in Table 1 displayed dry, cauliflower-like colonies when grown on BHI agar plates and formed flocculent suspensions within a liquid BHI medium (Figure 1). Comprehensive physiological and biochemical observations showed that all 10 strains exhibited positive results for catalase activity and nitrate reduction. They demonstrated the ability to utilize citrate as a carbon source but showed no utilization of mannitol, arabinose, or sorbitol. No strains displayed enzyme secretion capable of hydrolyzing gelatin, starch, or casein. The hydrolysis of esculin was observed solely in strains WL171012, LY21930, and WL22722, while the remaining strains demonstrated an inability to perform this hydrolysis (Table 4).

**Table 4:** Physiological and biochemical characteristics of 10 *N. seriolae* strains.

Items	Strain name										
	WL171012	LY20810	LY20910	LY21712	LY21811	LY21930	LY211019	LY211026	LY211129	WL22722	JCM3360T
*Enzymes activity*											
Catalase	+	+	+	+	+	+	+	+	+	+	+
Oxidase	−	−	−	−	−	−	−	−	−	−	−
Urease	−	−	−	−	−	−	−	−	−	−	−
Nitrate reduction	+	+	+	+	+	+	+	+	+	+	+
*Hydrolyzing activity*											
Aseculin	+	−	−	−	−	+	−	−	−	+	+
Gelatin	−	−	−	−	−	−	−	−	−	−	−
Starch	−	−	−	−	−	−	−	−	−	−	−
Casein	−	−	−	−	−	−	−	−	−	−	−
*Growth on sole carbon sources*											
Mannitol	−	−	−	−	−	−	−	−	−	−	−
Arabinose	−	−	−	−	−	−	−	−	−	−	−
Sorbitol	−	−	−	−	−	−	−	−	−	−	−
Citrate	+	+	+	+	+	+	+	+	+	+	+

“+” for positive, “−” for negative.

### 3.2 Evolutionary analysis of 16S rDNA sequences

**Figure 2:**
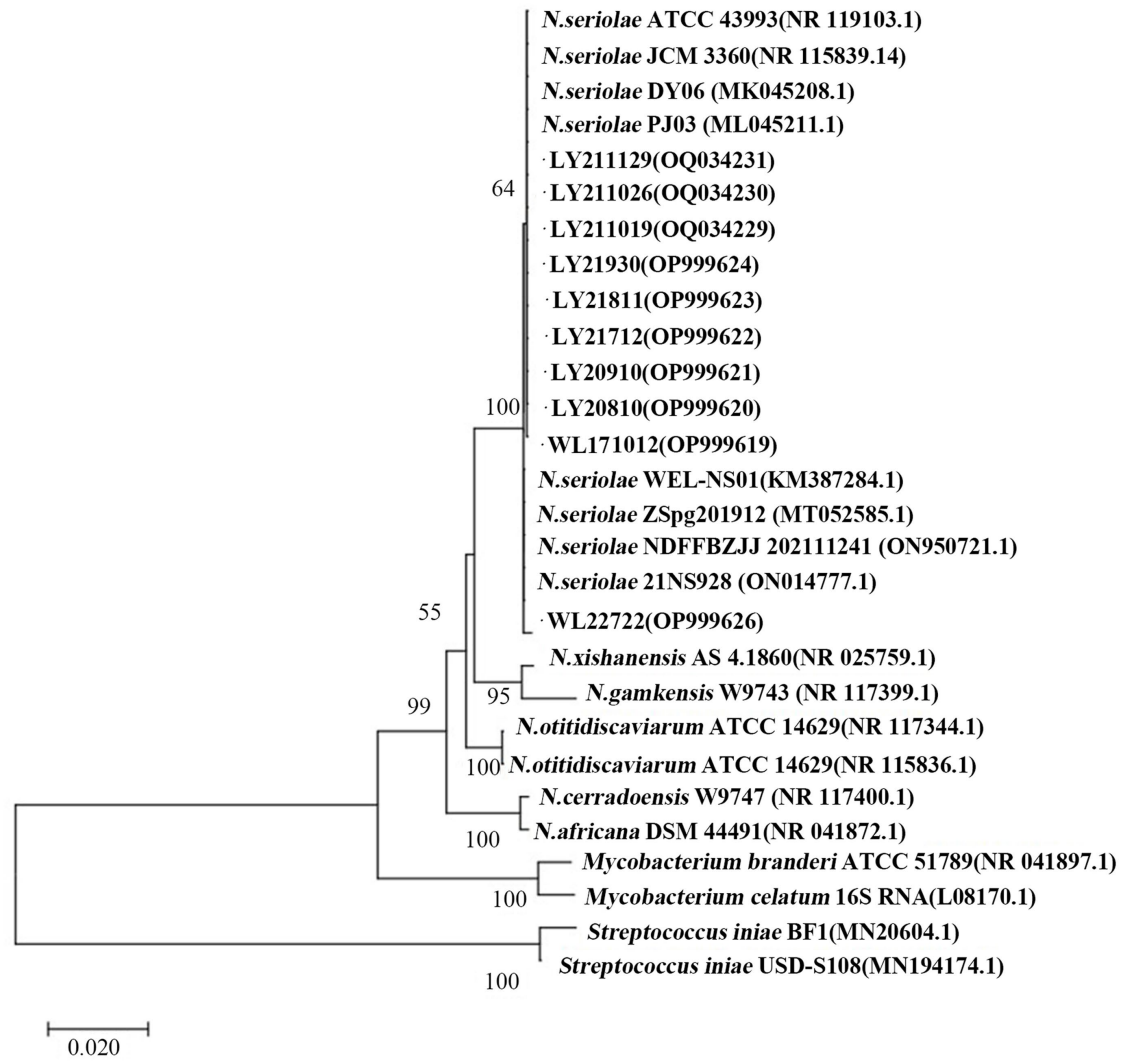
Construction of a phylogenetic tree by NJ method based on 16S rDNA gene of 10 *N. seriolae* strains and *Nocardia* reference strains. Numbers in parentheses represent the sequences accession number of each strain in GenBank; Scale “0.020” represents the unit of genetic distance.

**Figure 3:**
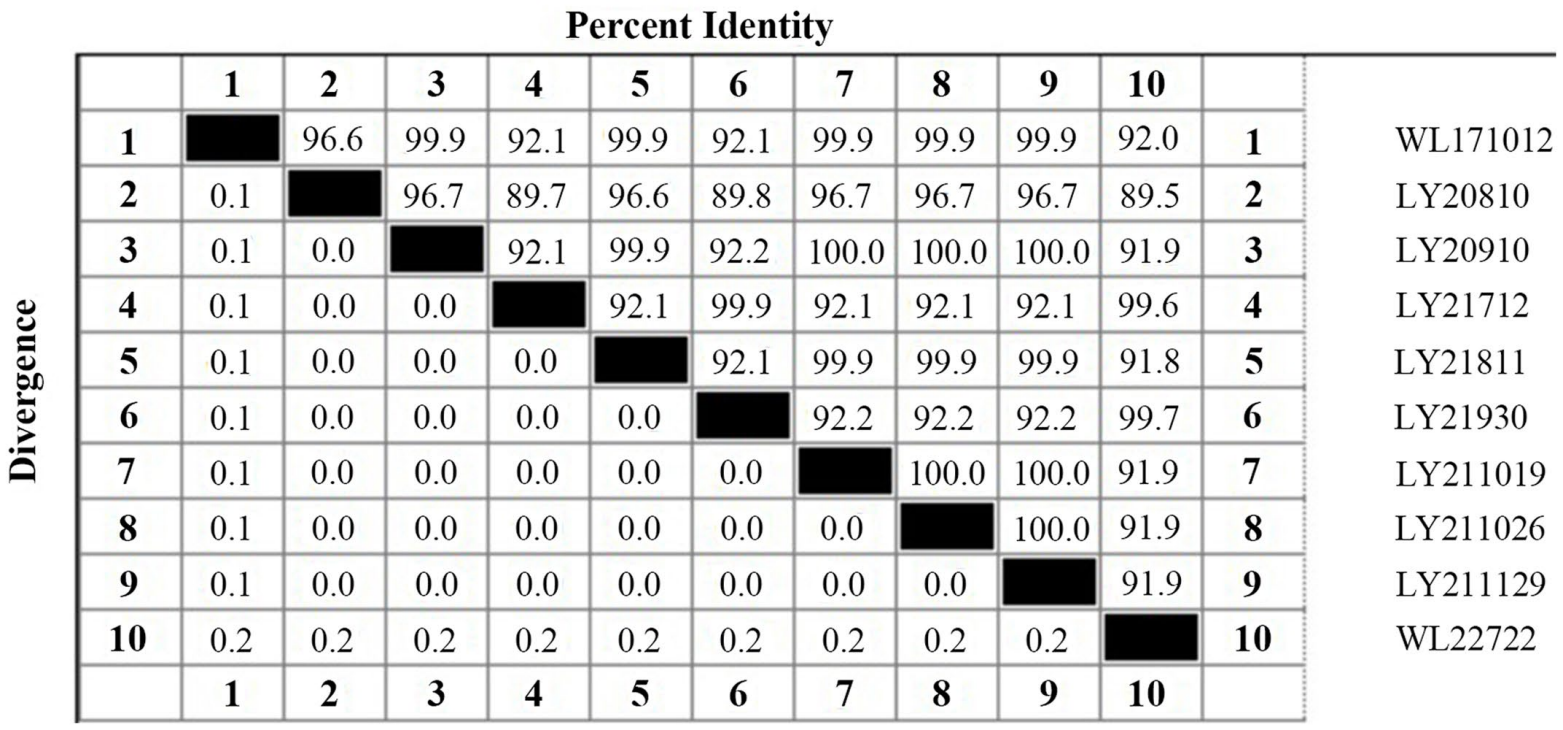
Percent identity and divergence of 16S rDNA gene of 10 *N. seriolae* strains.

The 16S rDNA genes from the 10 strains underwent amplification, sequencing, and subsequent Blastn comparison. Findings displayed a striking similarity range of 99.71 to 99.86% with *N. seriolae*. Notably, the phylogenetic tree analysis demonstrated a close clustering of these 10 strains alongside *N. seriolae*, highlighting a substantial affinity (Figure 2). Furthermore, the sequence similarity between the 10 strains varied from 92 to 100%, underscoring notable differences among distinct sources of *N. seriolae* strains (Figure 3). The GenBank accession numbers for the 16S rDNA gene sequences of bacterial strains WL171012, LY20810, LY20910, LY21712, LY21811, LY21930, LY211019, LY211026, LY211129, and WL22722 are OP999619–OP999624, OQ034229–OQ034231, OP999626, respectively.

### 3.3 Virulence genes

**Figure 4:**
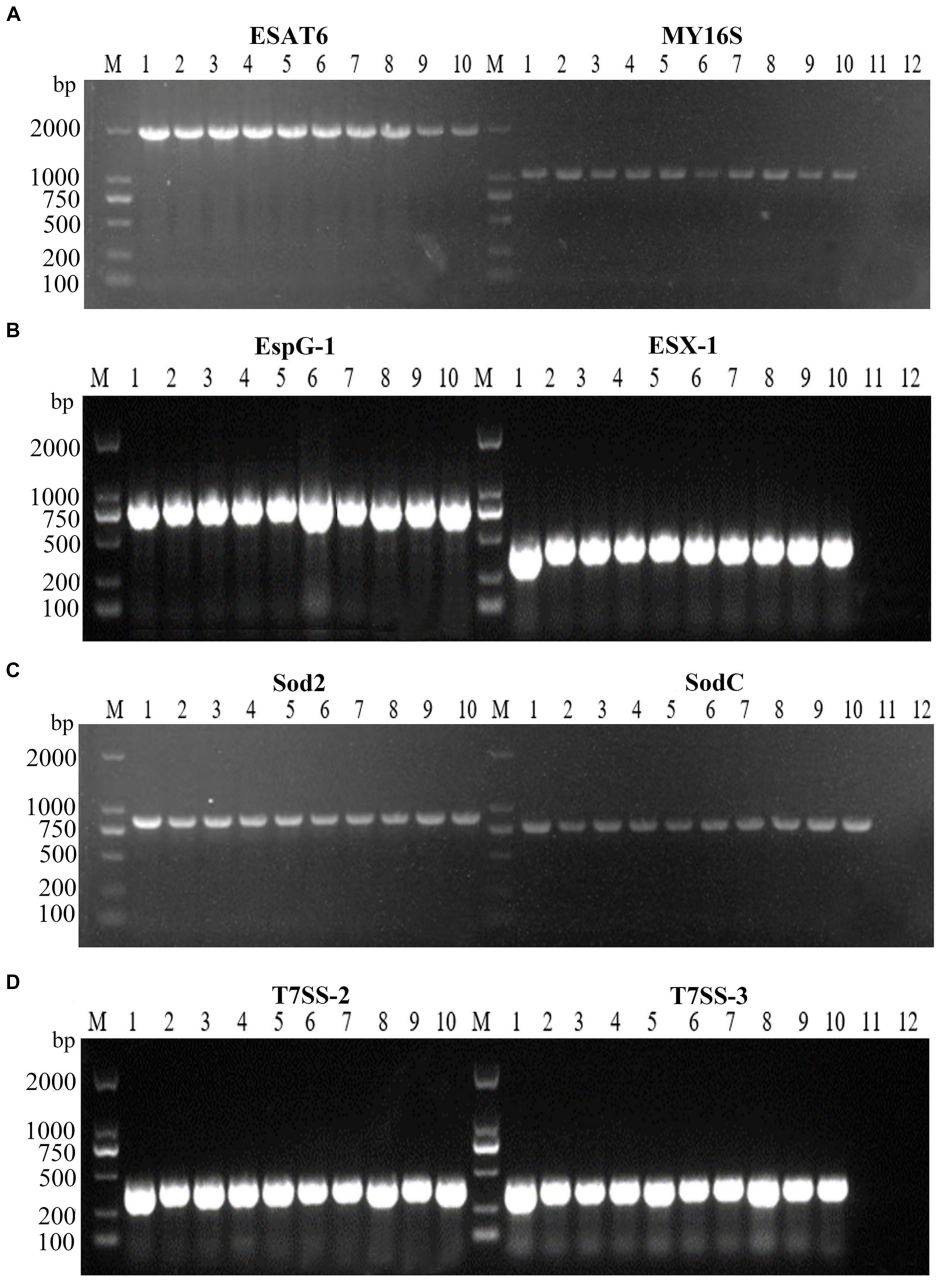
PCR amplifications result of virulence factors of 10 strains. M: D2000 Marker; 1: WL171012; 2: LY20810; 3: LY20910; 4: LY21712; 5: LY21811; 6: LY21930; 7: LY211019; 8: LY211026; 9: LY211129; 10: WL22722; **(A)**-11: ESAT6 negative control; **(A)**-12: MY16S negative control; **(B)**-11: EspG-1 negative control; **(B)**-12: ESX-1 negative control; **(C)**-11: Sod2 negative control; **(C)**-12: SodC negative control; **(D)**-11: T7SS-2 negative control; **(D)**-12: T7SS-3 negative control.

Virulence-associated genes, ESX-1, T7SS-2, T7SS-3, EspG1, sodC, sod2, and ESAT6, were identified via PCR analysis, and the findings indicated the presence of these seven virulence genes within all 10 tested strains of *N. seriolae* (Figure 4). Additionally, it was observed that the sequences of each gene exhibited a high degree of consistency across the strains.

### 3.4 Pathogenicity of the strains

**Figure 5:**
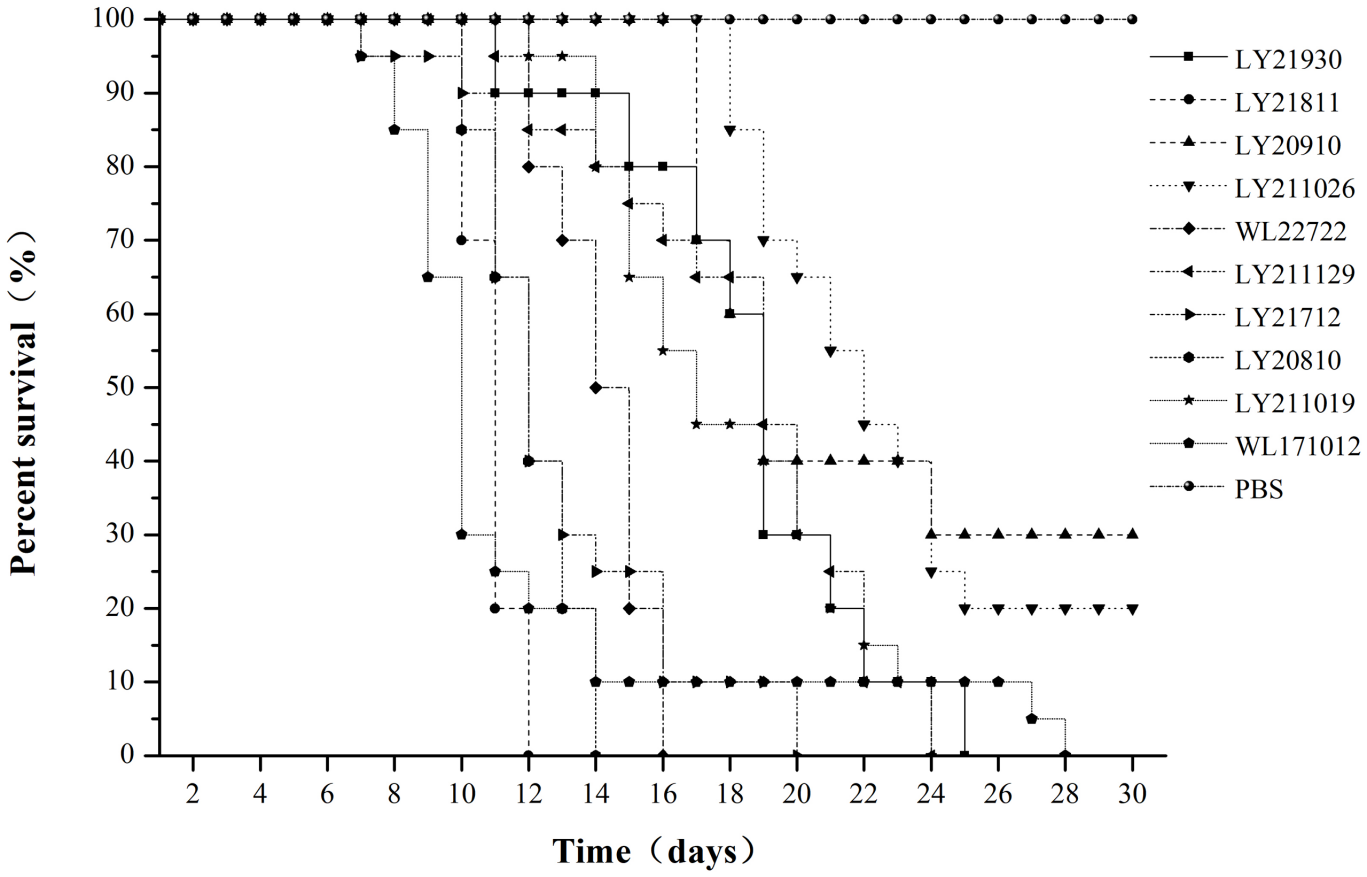
Survival rate of *M. salmoides* challenged with 10 strains of *N. seriolae* for 30 days.

Through challenge experiments revealed that after 30 days post-challenge, eight strains (WL171012, LY20810, LY21712, LY21811, LY21930, LY211019, LY211129, and WL22722) exhibited a mortality rate of 100% in largemouth bass. LY20910 and LY211026 had mortality rates of 70 and 80%, respectively. The control group exhibited a survival rate of 100%. Among them, strains LY21811, LY20810, WL22722, and LY21712 showed a mortality rate of 100% within 20 days. LY21811 exhibited the highest virulence, with a mortality rate of 100% within 12 days, while LY20910 demonstrated the lowest virulence (Figure 5).

**Figure 6:**
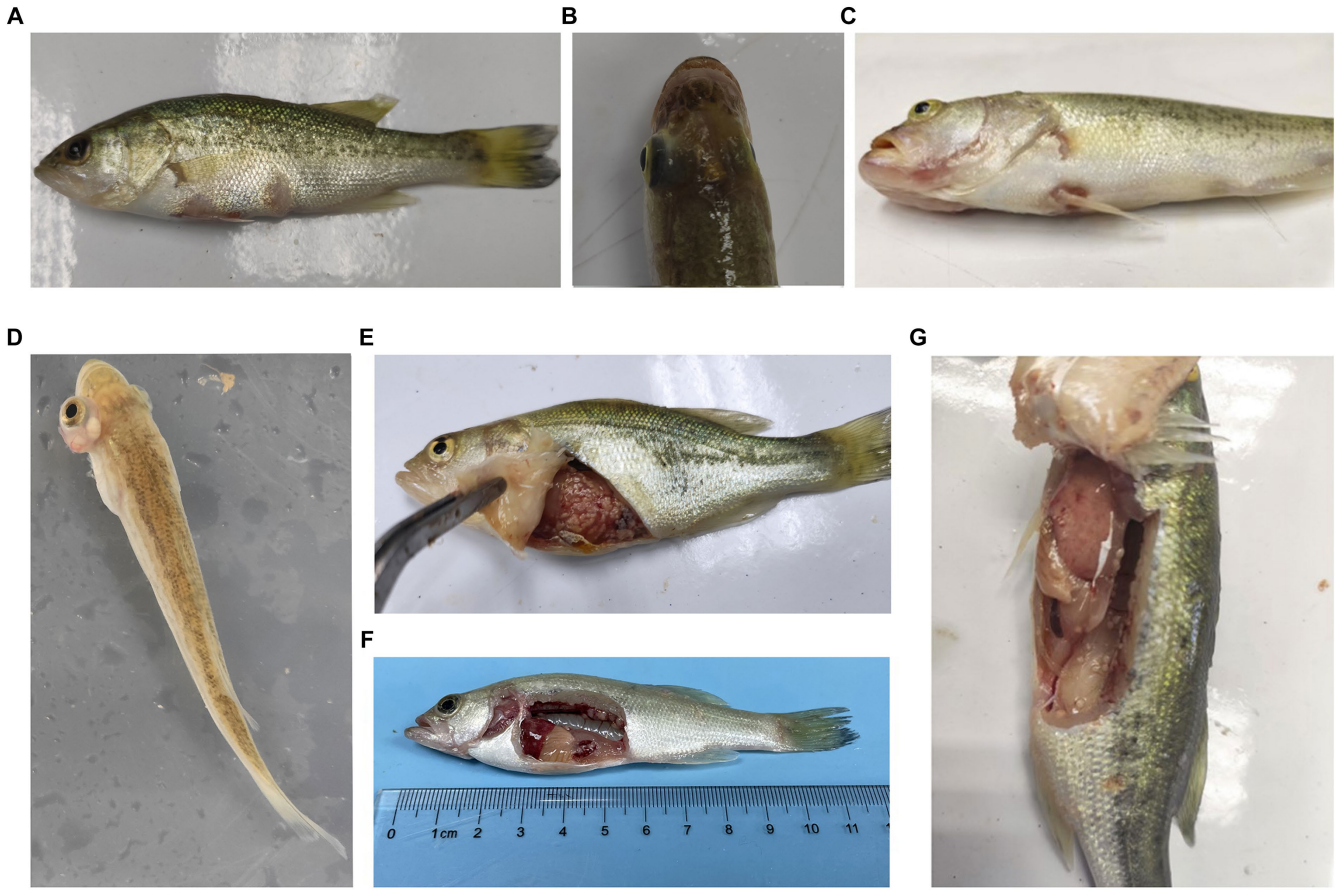
Typical symptoms of *M. salmoides* during the pathogenicity testing. **(A)** Ulcer focal in the skin; **(B)** brain flush red; **(C)** lips flush red; **(D)** exophthalmos and swelling; white nodules in **(E)** abdominal tissue, **(F)** kidney **(G)** peritoneum.

During the challenge experiments, similar clinical symptoms were observed among infections caused by the 10 bacterial strains. Infected fish exhibited surface redness, swelling, and ulceration, with red patches around the mouth. Additionally, numerous white nodules were observed within organs such as the liver, spleen, kidneys, mesentery, and peritoneum (Figure 6).

### 3.5 Drug sensitivity

In order to provide clinically relevant guidance, strain LY21811, which exhibited the highest pathogenicity in largemouth bass, was selected for sensitivity testing against 16 commonly used antibiotics. The results revealed that this strain displayed high sensitivity to azithromycin, doxycycline, flumequine, compound sulfamethoxazole, gentamicin, rifampicin, neomycin, kanamycin, and clindamycin. It showed moderate sensitivity to enrofloxacin and novobiocin but demonstrated resistance to β-lactam antibiotics (ceftriaxone, amoxicillin, cefotaxime, trimethoprim-sulfamethoxazole and penicillin).

The results of sensitivity testing on phytochemicals indicated that all 10 drugs (0.5 mg/mL) exhibited antibacterial effects. Among them, tannic acid showed the most significant inhibitory effect, followed by magnolol and coumarin (Table 5). Further determination of the inhibitory concentration of tannic acid against strain LY21811 revealed the minimum inhibitory concentration to be 0.0625 mg/mL (Table 6).

**Table 5:** *In vitro* antibacterial effects of 10 drugs on strain LY21811.

Drug name	Inhibitory zone (mm)
Quercetin	8.33 ± 0.56
Tannic acid	30.67 ± 3.60
Epicatechin	19.67 ± 1.53
Coumarin	27.33 ± 2.08
Magnolol	28.33 ± 0.57
Matrine	11.33 ± 1.15
Quercetin-3-O-glucoside	9.83 ± 0.29
Glycyrrhizic acid	15.50 ± 1.50
Baicalin	24.93 ± 1.38
Forsythoside B	22.00 ± 3.00

**Table 6:** Antibacterial effect of tannic acid with different concentrations on strain LY21811.

Tannic acid concentration (mg/mL)	Inhibitory zone (mm)
0.50	29.00 ± 2.64
0.25	15.30 ± 1.15
0.125	11.60 ± 0.65
0.0625	9.16 ± 0.28
0.0313	0

## 4 Discussion

Members of the genus *Nocardia* are Gram-positive, non-motile, and aerobic actinomycetes, belonging to the family Nocardiaceae. They are widely distributed in various environments such as air, soil, activated sludge, water, decomposing vegetation, animal excreta, and human tissues (27). This genus contains more than 90 recognized species and is widely distributed in both aquatic and terrestrial habitats (28). In the early phases of categorizing, the classification of *Nocardia* relied mainly on their observable physical and biochemical characteristics (29, 30). However, modern methods, including DNA sequencing and genomic analysis, have provided a more accurate and comprehensive understanding of the relatedness among different *Nocardia* species (11, 31–33). This study employed both phenotypic and molecular classifications for the isolated strains.

In this study, 10 bacterial strains were isolated from samples of *M. salmoides* and *C. argus* affected by “nodular disease” collected from aquaculture farms various regions in Zhejiang Province, China, between 2017 and 2022. During cultivation process, these strains exhibited slow growth after revival, taking 5–7 days at 28°C to form white or pale yellow colonies with grainy or petal-like appearances. The colonies were rough, fragile with irregular edges, and displayed raised folds or wrinkles, consistent with previous literature reports (34, 35). Phylogenetic analysis based on the 16S rDNA gene sequences revealed that eight strains sharing 99% homology with a strain isolated from *M. salmoides* in Sichuan (MK045211.1) and the type strain *N. seriolae* ATCC43993. Furthermore, strains WL171012 and WL227222 were found to cluster with *N. seriolae* isolated from *M. salmoides* in Sichuan (KM387284.1) and silver pomfret in Zhejiang (MT052585.1). On the other hand, strains LY20910, LY211019, LY211026, and LY211129 displayed 100% similarity. Notably, LY211019, LY211026 and LY211129 were isolated from different aquaculture farms in Deqing of Huzhou, may belong to the same strain type. These findings suggest the presence of genetic polymorphism among strains of the same species across similar or distinct environments, possibly attributed to substantial intraspecific diversity within *N. seriolae*. It is noteworthy that different isolates from diverse aquaculture farms might also share a common genotype.

Previous studies have shown the factors such as NlpC/P60, superoxide dismutase (SOD), and glutaminyl cyclase GluNS gene in the pathogenicity of *N. seriolae* (19, 20). SOD has been identified as a virulence factor, as it is a secreted protein capable of inducing apoptosis in FHM cells (19). The Type VII secretion system (T7SS) is responsible for the secretion of virulence proteins in mycobacteria, and the infection of pathogenic mycobacteria to hosts is mediated by the virulence factors secreted through T7SS (21, 22). Proteins ESAT-6 and EspG in the ESX-1 secretion system of *Mycobacterium tuberculosis* are important virulence factors. Their absence inhibits the growth and replication of *M. tuberculosis* in macrophages, leading to the attenuation of *M. tuberculosis* virulence (23). In this study, the seven known virulence genes were all detected in isolated bacterial strains, and the identified sequences are basically the same. Moreover, mortality rates of largemouth bass caused by these strains ranged from 70 to 100%, which suggested that these virulence genes play crucial roles in pathogen invasion, and further elucidation of these mechanisms is essential for future investigations.

Wang et al. (9) reported that 100% of snakeheads challenged with *N. seriolae* at 1.16 × 10^6^ CFU per fish succumbed within 18 days, whereas 95% of striped tigerfish challenged with 1.2 × 10^7^ CFU per fish died within 5 weeks (36). Similarly, all snubnose pompano infected with 1.3 × 10^6^ CFU per fish perished within 10 days (37). In contrast, eels exhibited higher resistant to infection, with only a 20% mortality rate observed after a challenge with 1.8 × 10^7^ CFU per fish over 4 weeks (3). In this study, strains LY21811, LY20810, and WL22722 caused 100% mortality within 16 days, while LY21712, WL171012, LY21930, LY211019, and LY211129 led to 100% mortality between 20–28 days. Strains LY20910 and LY211026 showed mortality rates of 70 and 80%, respectively, on the 30th day. Notably, the strains WL171012 and WL22722 isolated from diseased *C. argus* exhibited a top-three mortality rate in largemouth bass, indicating strong cross-host pathogenicity of *N. seriolae* and suggesting the potential for higher virulence in strains originating from *C. argus*. The various strains investigated in this study showed high virulence in largemouth bass, highlighting largemouth bass as a highly susceptible host to *N. seriolae*. After regression infection with the 10 strains, largemouth bass displayed similar clinical signs and typical nodular symptoms to that observed in other cases of nocardiosis (3, 31, 36–38).

Similar to the causative agents of human nocardiosis, *N. seriolae* is regarded as an intracellular pathogen capable of invading and thriving within host cells, including phagocytes (12, 39, 40). The intracellular behavior of this bacterium presents challenges for disease control, possibly due to the formation of nodules obstructing the action of drugs on its internal bacteria. Presently, the primary treatment for *N. seriolae* in aquaculture involves antibiotic utherapy. However, the improper and prolonged use of antibiotics may lead to varying degrees of drug resistance among strains from different times and locations. Drug sensitivity tests revealed that LY21811 displayed resistance to amoxicillin and penicillin but susceptibility to fluoroquinolones, azithromycin, and doxycycline, similar to the previous findings (41). Considering the concern of antibiotic resistance in *N. seriolae*, it becomes imperative to explore suitable antibiotic alternatives to manage infections caused by this pathogen.

Phytochemicals with antibacterial and anti-inflammatory effects, such as quercetin, matrine, tannic acid, magnolol, epicatechin, baicalin, and coumarin, were selected in this experiment to verify their ability to inhibit the growth of pathogenic bacteria, providing preventive and therapeutic effects against bacterial diseases in fish (42–45). In this study, we discovered that tannic acid, coumarin, and magnolol demonstrate substantial inhibitory effects against LY21811, with tannic acid exhibiting the most significant efficacy. Tannic acid, the principal active component found in gallnuts, can impact the growth metabolism of microorganisms by causing coagulation of the protoplasm and various enzymes within the pathogen, and it has been primarily studied and reported for its role in preventing and treating bacterial diseases in fish farming (46–48). Considering these findings with our results, the application of plant-derived medications like tannic acid is highly probable to emerge as an effective and safe treatment approach for nocardiosis infections in largemouth bass.

## Data availability statement

Original datasets are available in a publicly accessible repository: The original contributions presented in the study are publicly available. This data can be found here: https://www.ncbi.nlm.nih.gov/nucleotide, accession numbers: OP999619, OP999620, OP999621, OP999622, OP999623, OP999624, OP999626, OQ034229, OQ034230, OQ034231.

## Ethics statement

The animal study was approved by Ethics Committee of Ningbo Sansheng Biological Technology Co., Ltd. (approval code: NBSS-AEC-202208002). The study was conducted in accordance with the local legislation and institutional requirements.

## Author contributions

WZ: Investigation, Methodology, Writing – original draft, Writing – review & editing. KZ: Writing – original draft. LH: Formal analysis, Writing – review & editing. NY: Writing – review & editing. LL: Software, Validation, Writing – review & editing. LC: Funding acquisition, Project administration, Writing – review & editing. JY: Investigation, Resources, Writing – review & editing. MD: Supervision, Writing – review & editing. JS: Validation, Writing – review & editing. XP: Project administration, Resources, Writing – review & editing.
